# Insights into the Membranolytic Activity of Antimalarial Drug-Cell Penetrating Peptide Conjugates

**DOI:** 10.3390/membranes11010004

**Published:** 2020-12-22

**Authors:** Luísa Aguiar, Marina Pinheiro, Ana Rute Neves, Nuno Vale, Sira Defaus, David Andreu, Salette Reis, Paula Gomes

**Affiliations:** 1 LAQV-REQUIMTE, Departamento de Química e Bioquímica, Faculdade de Ciências, Universidade do Porto, Rua do Campo Alegre 687, P-4169-007 Porto, Portugal; luisa.silva@fc.up.pt; 2 LAQV-REQUIMTE, Departamento de Ciências Químicas, Faculdade de Farmácia, Universidade do Porto, Rua de Jorge Viterbo Ferreira 228, P-4050-313 Porto, Portugal; marinabppinheiro@gmail.com (M.P.); shreis@ff.up.pt (S.R.); 3 Centro de Química da Madeira, Universidade da Madeira, Campus da Penteada, Ilha da Madeira, P-9020-105 Funchal, Portugal; rutepneves@gmail.com; 4 OncoPharma Research Group, Center for Health Technology and Services Research (CINTESIS), Rua Dr. Plácido da Costa, P-4200-450 Porto, Portugal; nunovale@med.up.pt; 5 Faculdade de Medicina, Universidade do Porto, Alameda Prof. Hernâni Monteiro, P-4200-319 Porto, Portugal; 6 Proteomics and Protein Chemistry Group, Department of Experimental and Health Sciences, Pompeu Fabra University, Dr. Aiguader 88, E-08003 Barcelona, Spain; sira.defaus@upf.edu (S.D.); david.andreu@upf.edu (D.A.)

**Keywords:** antimalarial, biophysics, cell penetrating peptide, dynamic light scattering, haemolysis, lipid membranes, surface plasmon resonance

## Abstract

Conjugation of TP10, a cell-penetrating peptide with intrinsic antimalarial activity, to the well-known antimalarial drugs chloroquine and primaquine has been previously shown to enhance the peptide’s action against, respectively, blood- and liver-stage malaria parasites. Yet, this was achieved at the cost of a significant increase in haemolytic activity, as fluorescence microscopy and flow cytometry studies showed the conjugates to be more haemolytic for non-infected than for *Plasmodium*-infected red blood cells. To gain further insight into how these conjugates distinctively bind, and likely disrupt, membranes of both *Plasmodium*-infected and non-infected erythrocytes, we used dynamic light scattering and surface plasmon resonance to study the interactions of two representative conjugates and their parent compounds with lipid model membranes. Results obtained are herein reported and confirm that a strong membrane-disruptive character underlies the haemolytic properties of these conjugates, thus hampering their ability to exert selective antimalarial action.

## 1. Introduction

Malaria is unquestionably the most devastating parasitic disease worldwide, with a severe impact on at both health and economic levels; in 2018 alone, over 400,000 people died from this disease—mainly young children—and almost 230 million cases of malaria were reported [1]. While great progress has been made in reducing the burden of malaria, namely through the wide use of insecticide-treated mosquito nets for malaria prevention and the use of artemisinin-based combination therapy for treatment of infections, growing resistance to past and present antimalarial drugs (AM) still requires continued research to stay one step ahead [2]. This is a difficult challenge, considering the complexity of the malaria parasite, whose lifecycle in the human host comprises different developmental exo- and intra-erythrocytic stages, each of which involves significant changes to the host cells upon parasite invasion. For instance, right after invading the erythrocyte (RBC), the parasite is isolated from the cytoplasm of host cells by the parasitophorous vacuole (PV) membrane and undergoes continuous development through ring forms, trophozoites and schizonts, culminating in differentiation into 10–20 merozoites [3]. RBC remodeling begins instantly after invasion by the parasite, through the expression and exportation of several hundreds of proteins required to assemble a molecular machinery in the host cell, which allows for protein trafficking, harvesting of nutrients and mechanisms to evade host immune responses [4,5]. This process entails distinct morphological and biochemical alterations in the host cell membrane [6], including a marked increase in the fluidity of the RBC membrane [7], whose lipid fatty acid composition is altered [8], as well as increased permeability through newly formed membrane pores [5,9,10,11]. Other significant alterations include phospholipid composition and transbilayer distribution [10]. The membrane of RBC infected with late-stage schizonts contain up to five times more phospholipids than non-infected RBC (niRBC) [3,9]. Specifically, the lipid domain of niRBC is composed of virtually equal parts of protein and lipid, with the majority of lipids consisting of cholesterol and phospholipids. While cholesterol is equally distributed between the two halves or leaflets of the lipid bilayer, the other lipids are asymmetrically distributed: glycolipids, phosphatidylcholine (PC), and sphingomyelin (SM) are located in the outer leaflet; whereas phosphatidylinositol (PI), phosphatidylethanolamine (PE), and phosphatidylserine (PS) are found in the inner leaflet, facing the cytoplasm [12]. The development of *Plasmodium* parasites inside RBC results in partial loss of the normal asymmetry of the membrane’s phospholipids, with increased amounts of PE and PS and a decreased amount of PC in the exoplasmic (outer) leaflet of infected cells. These alterations in *Plasmodium*-infected RBC (PiRBC) are gradual and depend upon the stage of parasite development: cells infected with early stages show a partial redistribution of PE and PC but not PS, while cells infected with late stages present an additional redistribution of PS [6,10,13,14]. Additionally, parasite growth is accompanied by a depletion in SM content (~47%) and cholesterol/phospholipid ratio (~55%), plus an increase in membrane fluidity [7].

**Figure 1:**
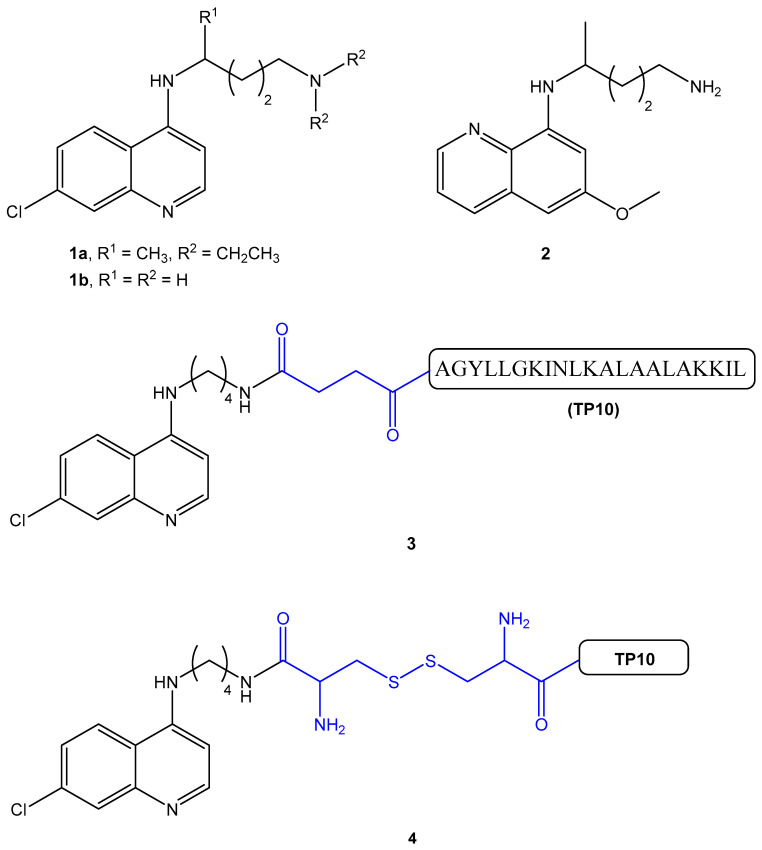
Structures of the antimalarial drugs blood-schizonticide chloroquine (CQ) (1) and gametocytocide primaquine (PQ) (2), and of the CQ-TP10 conjugates CQ-C4-TP10 (3) and CQ-S-S-TP10 (4). The full amino acid sequence (in single letter code) of TP10 is shown for conjugate 3 [24].

Recently, considering the potential value of previously reported antimalarial peptides [15,16,17], we have addressed the covalent coupling of known AM to different cell-penetrating peptides (CPPs), some of which possess intrinsic antimalarial properties, e.g., TP10 [15,18,19]. Thus, preparation of AM-CPP conjugates derived from two AM with complementary antimalarial action, namely, the well-known blood-schizonticide chloroquine (CQ, 1a in Figure 1) and the classical tissue-schizonticide and gametocytocide primaquine (PQ; 2, Figure 1) was envisaged. In the case of CQ conjugates, the primary amine analogue of CQ (1b in Figure 1) had to be used instead of CQ itself, to ensure the coupling [18]. As a result of AM coupling, the activity of the different CPPs was generally improved, with AM-TP10 conjugates standing out as the most potent of the tested set [18,19]. Yet, the AM-TP10 conjugates were found to be substantially more haemolytic than their parent building blocks, and haemolysis by CQ-TP10 was observed to be more extensive on niRBC than on PiRBC [18]. In other words, it appears that, upon coupling to AM, the original cell-penetrating ability of TP10 is converted into a cell-disruptive character that is more pronouncedly exerted onto niRBC than onto PiRBC. In view of these puzzling observations and considering the aforementioned differences between the membranes of unparasitized and parasitized RBC, we have investigated the interactions between two representative AM-TP10 conjugates (3 and 4, Figure 1) and large unilamellar vesicle (LUV) models of niRBC and PiRBC membranes. LUV composed by zwitterionic 1,2-dimyristoyl-sn-glycero-3-phosphocholine (DMPC) or 1-palmitoyl-2-oleoyl-sn-glycero-3-phosphocholine (POPC) were selected as simplistic mimics of niRBC membranes; these models are typically used to simulate the neutral outer leaflet of eukaryotic cells, which are mainly composed of phosphatidylcoline (PC) headgroups [20]. In turn, LUV composed by 1,2-dimyristoyl-sn-glycero-3-phosphorylglycerol (DMPG) or 1-palmitoyl-2-oleoyl-sn-glycero-3-phosphoglycerol (POPG) were used as simplistic models for PiRBC, as they represent an increased amount of negatively charged phospholipids present in the outer leaflet of PiRBC [21,22,23]. Results obtained are herein reported and discussed.

## 2. Results

The AM-CPP conjugates shown in Figure 1, CQ-C4-TP10 (3) and CQ-S-S-TP10 (4), were selected based on their different in vitro antimalarial and haemolytic activities [18]. Their parent building blocks, TP10 and CQ, were included in the study in order to make a comparative assessment of their behavior while separate individual molecules and when covalently bound to each other. Biophysical interactions between test compounds and zwitterionic/anionic lipid model membranes were investigated by surface plasmon resonance (SPR) and dynamic light scattering (DLS), as follows.

### 2.1. SPR Analysis of TP10 and Conjugates Binding to Model Membranes

Binding of TP10 and its CQ conjugates to POPC and POPG membrane models of, respectively, niRBC and PiRBC, was monitored using real-time SPR analysis. The parent drug, CQ, was excluded in this analysis as SPR could be unreliable for monitoring binding affinities of low molecular weight ligands [25].

**Figure 2:**
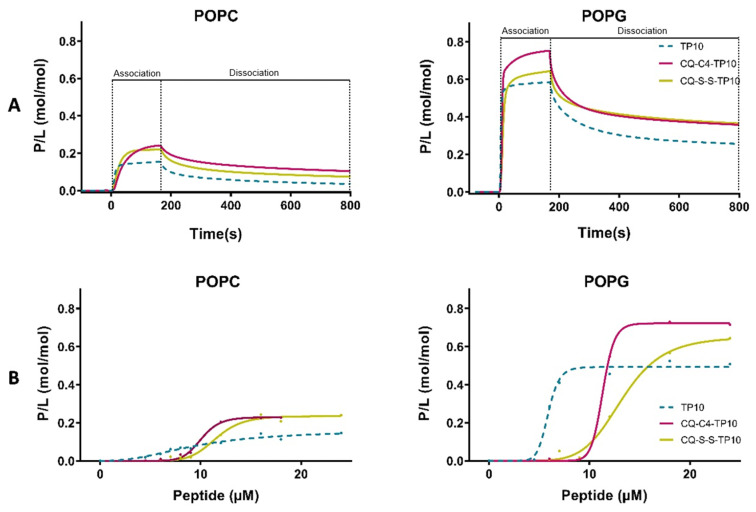
Binding of TP10 and CQ-TP10 conjugates to lipid model membranes as monitored using surface plasmon resonance (SPR). (**A**) Sensorgrams obtained upon injection of 16 µM TP10 (dotted lines) and its CQ conjugates over 1-palmitoyl-2-oleoyl-sn-glycero-3-phosphocholine (POPC) or 1-palmitoyl-2-oleoyl-sn-glycero-3-phosphoglycerol (POPG) bilayers deposited onto an L1 chip surface. Analytes were injected for 180 s (association phase) and lipid dissociation was monitored for 600 s (dissociation phase). Signal was normalized to peptide-to-lipid ratio (P/L; mol/mol) as detailed in Materials and Methods; (**B**) Dose-response curves for each lipid bilayer. P/L obtained at the end of the association phase (t = 180 s) was plotted as a function of peptide concentration in solution.

**Figure 3:**
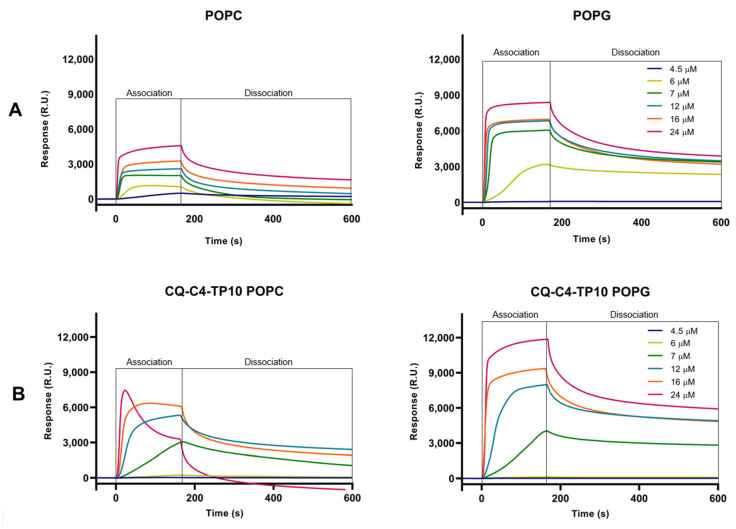
SPR sensorgrams obtained for (**A**) TP10 and (**B**) CQ-C4-TP10 binding to POPC and POPG model membranes at six different concentrations ranging from 4.5–24 μM. While the interaction of increasing concentrations of CQ-C4-TP10 with POPG membranes results in a typical sensorgram (as does TP10 upon interaction with both types of membranes), the same does not happen for POPC membranes: at 16 µM, slight POPC membrane disruption is observed as a decrease in response units (RU) during the association phase; at 24 µM, it is possible to observe that this conjugate acts like a “detergent”, disrupting the zwitterionic membranes and removing them from the sensor chip (negative RU).

Sensorgrams obtained in Figure 2 clearly show the preferential binding of both TP10 and its CQ conjugates to anionic POPG membranes, as expected from the electrostatic interaction between the positively charged CPP and the negatively charged membranes. Relevantly, SPR results also showed that CQ-C4-TP10 and CQ-S-S-TP10 bind more strongly to neutral POPC membranes than the parent CPP TP10 alone, suggesting that CQ plays an important role in the establishment of conjugate interactions with the zwitterionic membranes. Moreover, the most haemolytic of the test compounds, conjugate CQ-C4-TP10, was found to disrupt POPC but not POPG lipid bilayers at 24 µM (Figure 3), whereas the same was observed for CQ-S-S-TP10 but only at 32 µM (data not shown). In contrast, the free parent peptide TP10 did not disrupt any of the lipid bilayers in the full concentration range used in the study. SPR data are consistent with the haemolytic activity formerly reported by us, whereby CQ-C4-TP10 was found to be considerably more haemolytic towards niRBC than CQ-S-S-TP10 or TP10 [18].

### 2.2. Effects of CQ-TP10 Conjugates on the Size of the Lipid Vesicles

**Figure 4:**
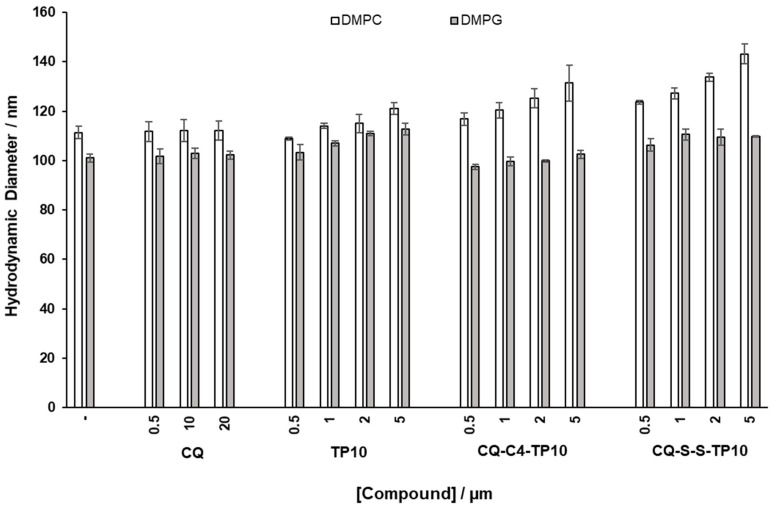
Effects of incubation with increasing concentrations of the test compounds on hydrodynamic diameter (HD) of zwitterionic 1,2-dimyristoyl-sn-glycero-3-phosphocholine (DMPC) and 1,2-dimyristoyl-sn-glycero-3-phosphorylglycerol (DMPG) liposomes, taken as simplistic models of niRBC and PiRBC, respectively.

**Figure 5:**
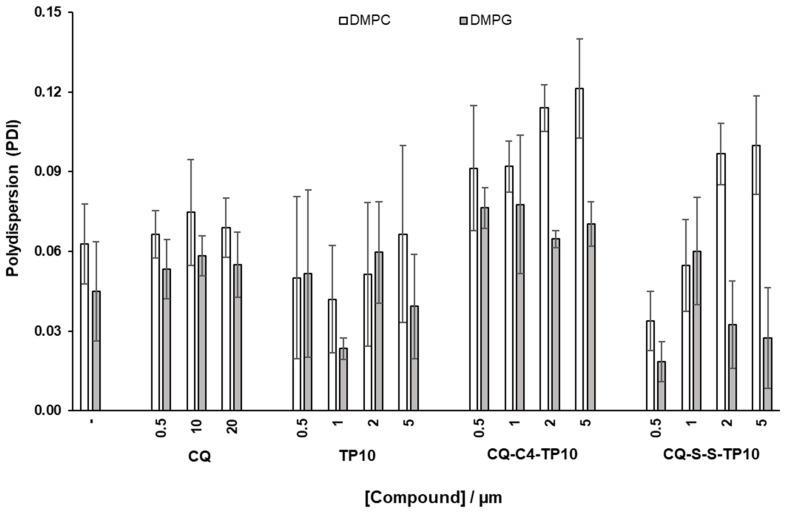
Effects of incubation with increasing concentrations of the test compounds on (polydispersion) PDI of DMPC and DMPG liposomes, taken as simplistic models of niRBC and PiRBC, respectively.

The average dimension and polydispersity (size distribution) of liposomes are relevant parameters for their characterization [26], as they are indicative of liposome stability and, as such, may afford a qualitative assessment of the interactions between model membranes and relevant test compounds. As such, DMPC and DMPG vesicles with a diameter of 100 nm were prepared and incubated for 30 min, at 37 °C, with increasing concentrations of CQ (1), CQ-C4-TP10 (3), CQ-S-S-TP10 (4), and TP10. The hydrodynamic diameter (HD) and polydispersion (PDI) of the vesicles were then assessed by DLS (Figure 4 and Figure 5, respectively). Results clearly show that the parent antimalarial drug, CQ, does not promote any size variations in either DMPG or DMPC liposomes, as no significant changes in HD or PDI were detected. Moreover, increasing concentrations of TP10 resulted in an increment on the size (HD) of both DMPG and DMPC vesicles, while the PDI remained relatively low, which strongly suggests that peptide merely accumulated on the surface of the vesicles without disrupting them. In clear contrast with these observations, both CQ-TP10 conjugates 3 and 4 displayed distinct behavior towards the two different lipid model membranes: while increasing concentrations of both CQ-TP10 conjugates did not promote any significant changes in DMPG vesicles, they did induce an increase in the size of DMPC liposomes, accompanied by a large increase in PDI, which may ultimately be responsible for the observed changes in the HD; this increase in polydispersion suggests vesicle aggregation and is in agreement with the observed toxicity of these conjugates towards niRBC [18]. Additionally, these observations correlate with SPR data, which show that this conjugate promotes the disruption of zwitterionic membranes.

### 2.3. Effect of CQ-TP10 Conjugates on the Fluidity of Model Membranes

**Figure 6:**
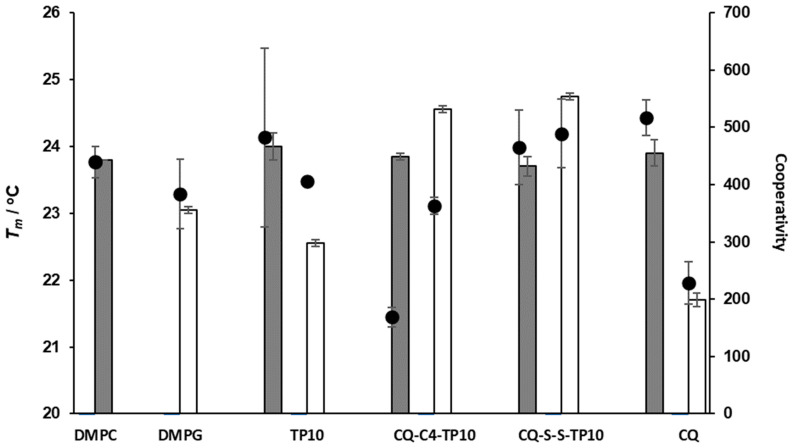
Main phase transition temperature (*T_m_*, bars) and cooperativity (B, dots) of DMPC and DMPG liposomes alone and in the presence of CQ (at 20 µM), TP10, CQ-C4-TP10, and CQ-S-S-TP10 (at 5 µM).

The main phase transition temperature (*T_m_*), i.e., the temperature at which the lipid vesicle undergoes the transition between the gel and fluid phases, constitutes another relevant parameter that can be derived by DLS, using the count rate of each measurement [27]. This parameter is closely related to the packing of membrane phospholipids, which can be altered by the addition of test compounds. Hence, to further investigate how CQ-TP10 conjugates 3 and 4 affected the *T_m_* of DMPC and DMPG vesicles, these were incubated with each of the conjugates and also with each of their parent building blocks, CQ and TP10, for 30 min at 37 °C; then, *T_m_* and cooperativity were determined by DLS, with temperatures ranging from 13 to 33 °C in each experiment. Data obtained are depicted in Figure 6 and show that neither compound seems to significantly affect the *T_m_* of DMPC liposomes. However, cooperativity clearly decreases when these vesicles interact with conjugate **3**, CQ-C4-TP10, suggesting that this particular conjugate interferes with the cooperative region of the membrane, possibly interacting with the first methylene groups of the alkyl chains and near the highly ordered polar head group, thus preventing some of the lipid molecules from participating in the melting transition [28,29]. This is a relevant observation, since conjugate 3 was previously found to be more toxic to RBC than its analogue CQ-S-S-TP10 (4) [18]. In turn, the *T_m_* of DMPG vesicles was considerably affected by all the test compounds, suggesting that these are interacting with the polar region of the bilayer, responsible for the packing of phospholipids. This was not surprising, as the test compounds are positively charged at pH 7.4 and hence would interact more strongly with the headgroups of negatively charged phospholipids. While TP10 and CQ-C4-TP10 did not alter the cooperativity of the main phase transition, suggesting that they interacted with the more superficial area of the lipids, the opposite was observed for CQ-S-S-TP10 and CQ: the induced changes in the cooperativity of DMPG vesicles suggested that these compounds interacted with the alkyl chains close to the headgroups (C1-C9) [29]. Interestingly, these compounds have a smaller impact on the size and polydispersion of DMPG vesicles, which is in agreement with their lower toxicity towards PiRBC versus niRBC [18].

## 3. Discussion

Data herein reported show that CQ-TP10 conjugates not only bind more strongly than the parent CPP to both DMPG and DMPC vesicles, but are also highly disruptive (especially, CQ-C4-TP10) to the latter, despite having higher binding affinity to the former. These findings are not as startling at they might seem at a first glance, considering the cationic nature of both TP10 and its CQ conjugates and, consequently, their expected stronger interaction with DMPG than with DMPC membranes. This is indeed observed: all the cationic peptide-based compounds do bind with higher affinity to DMPG vesicles than to DMPC ones. Yet, such interactions do not involve any significant damage, whereas CQ-TP10 conjugates clearly damage DMPC vesicles. This strongly suggests that the hydrophobic and planar quinoline core of CQ is able to insert deeper into the membrane of zwitterionic membranes, which, adding up to the cell penetrating ability of the peptide segment, results in a highly membrane-disruptive construct. This fully supports our earlier studies where the highest haemolytic effects were observed for CQ-TP10 conjugates, especially CQ-C4-TP10, on niRBC [18]. Arguably, the lipid membrane models herein used are an oversimplification of niRBC and PiRBC membranes, which are very complex mixtures of different lipids and also proteins and sugars, among others. Yet, the goal of this biophysical study was to answer more fundamental questions regarding the influence of the charge of the lipids (zwitterionic versus anionic) on the surface of the model membranes, considering that electrostatic interactions are fundamental in the first steps of a peptide-membrane interaction. Other factors providing useful insights into the unexpected lack of selectivity of CQ-TP10 conjugates for PiRBC should, however, be considered: firstly, the surface of both niRBC and PiRBC is negatively charged, due to the presence of several non-lipidic components [30]. It has been reported that glycophorins (proteins rich in negatively-charged sialic acid) present in the erythrocyte membrane surface are altered by an enzyme-like material released by the parasite upon invasion, to prevent further invasion by other parasites [9,31]. This results in a decrease in sialic acid at the outer leaflet of the membrane, hence possibly reducing the number of “hot spots” on which cationic CPP and their conjugates might bind through electrostatic interactions. In addition, positively charged knob-like protuberances (partly composed by histidine-rich proteins) emerge on the erythrocyte surface upon parasite entry [32], which might contribute to the same effect. In view of this, it is likely that CQ-TP10 conjugates display an even higher affinity with niRBC than that observed for their DMPC models, which, combined with the fact that CQ seems to promote a deeper insertion of the conjugate into the lipid bilayer, supports our previous findings from in vitro assays, namely, (i) strong haemolytic effects of CQ-TP10 conjugates on both PiRBC and, especially, niRBC, and (ii) lack of a potent and selective antimalarial action [18].

This work also highlights the scope and limitations of CPP conjugation as a strategy for the enhancement of the therapeutic action of small heterocyclic drugs, as it comes into agreement with recent literature on this topic. Recent reports on the conjugation of fluoroquinolones to TP10 show that this strategy resulted in significantly increased haemolytic action [33]. Thus, although TP10 is not highly haemolytic itself, the modification of this CPP with small heterocyclic moieties (e.g., fluoroquinolones [33], chloroquine, primaquine [18] or quinacrine—unpublished results) seems to considerably alter its mode of interaction with the membranes of RBC. Moreover, the conjugation strategy seems to have little impact on this interaction, although a more labile bond, such as a disulfide bridge, may contribute to a lesser haemolytic effect, likely due to its possible bioreductive cleavage [18].

## 4. Materials and Methods

### 4.1. Chemical Synthesis

Chemical synthesis of CQ (1b), peptide TP10, and CQ-TP10 conjugates 3 and 4 has been thoroughly described elsewhere by Aguiar et al. [18]. Briefly, 1b was synthesized via a nucleophilic aromatic substitution reaction between butane-1,4-diamine and 4,7-dichloroquinoline, following a previously reported in-house procedure [34]. In parallel, peptide TP10 was assembled by standard solid-phase peptide synthesis (SPPS) methods using the Fmoc/*^t^*Bu orthogonal protection scheme; to obtain the free parent peptide to be used as reference, it was cleaved from the resin by standard acidolysis with a trifluoroacetic acid (TFA)-based cocktail; in turn, to produce conjugates 3 and 4, additional modifications were carried out on-resin, prior to cleavage from the resin, as follows. For the synthesis of conjugate 3, compound 1b was further reacted with succinic anhydride in solution, to produce its *N*-carboxybutanoyl derivative that was next coupled on-resin to the *N*-terminus of the resin-bound TP10 sequence, using standard SPPS protocols; acidolytic cleavage with TFA delivered the target conjugate. To prepare conjugate 4, (i) compound 1b was reacted, via current in situ peptide coupling procedures, with a conveniently protected cysteine derivative, Boc-Cys(Trt)-OH; subsequent removal of the protecting groups with TFA delivered the *N*-cysteinyl derivative of 1b; (ii) in parallel, a suitably protected and side-chain activated cysteine derivative, Boc-Cys(Npys)-OH, was coupled to the *N*-terminus of the resin-bound TP10 sequence; acidolytic cleavage delivered Cys(Npys)-TP10 peptide, which was then reacted with the cysteine-modified derivative of 1b in solution to afford the target conjugate 4 via a thiol-disulfide exchange reaction. A more detailed description of all experimental procedures, as well as analytical and spectral data regarding all these compounds, can be found in [18].

### 4.2. Preparation of Liposomes

Lipids (DMPC, DMPG, POPC and POPG) were dissolved in chloroform/methanol (9:1 *v/v*), dried under a stream of nitrogen and left under reduced pressure overnight, yielding a dried lipid film. Phosphate buffer (0.05 M KH_2_PO_4_; 0.04 M NaOH; 0.1 M NaCl; pH = 7.4) was then added in order to obtain a final lipid concentration of 1200 µM, followed by vortexing at 37.0 ± 0.1 °C (well above the main phase transition temperature of the lipids) for 30 min, to generate a suspension of multilamellar vesicles (MLV). Unilamellar vesicles were prepared by extrusion of the MLV suspension through polycarbonate filters with a pore diameter of 600 nm (×10), 200 nm (×10) and, finally, 100 nm (for DMPC and DMPG) or 50 nm (for POPC and POPG), using a LIPEX Extruder, Northern Lipids Inc. (Burnaby, Canada). The size of the LUV obtained was confirmed through DLS in a Brookhaven Instrument (Holtsville, NY, USA).

### 4.3. Determination of the Hydrodynamic Diameter and PDI of the Liposomes

DMPC or DMPG vesicles (500 µM) were incubated with increasing concentrations of CQ (0,5, 10 and 20 µM), TP10, CQ-S-S-TP10 and CQ-C4-TP10 (0,5, 1, 2 and 5 µM) in phosphate buffer, for 30 min at 37 °C. Size of the vesicles was determined through DLS analysis.

### 4.4. Determination of Phase Transition Temperature and Cooperativity

The effects of CQ (20 µM), TP10, CQ-S-S-TP10 and CQ-C4-TP10 (5 µM) on the biophysical parameters (*Tm* and cooperativity) of DMPC and DMPG LUVs (500 µM) were determined by DLS, as described by Michel et al. [27]. The count rate was obtained using a BI-MAS DLS instrument (Brookhaven Instruments, Holtsville, New York, United States), equipped with a controlled temperature cell holder. The samples, prepared in phosphate buffer saline (PBS), were heated from 15.0 ± 0.1 °C to 35.0 ± 0.1 °C with intervals of 1.0 ± 0.1 °C and with an equilibration period of 2 min. At each temperature, 6 runs of 2 min were performed.

Data as the normalized mean count rate versus temperature are collected and fitted using Equation (1) [35]:

(1) $$r_{s}=r_{s1}+p_{1}T+\frac{r_{s2}-r_{s1}+p_{2}T-p_{1}T}{1+10^{B(\frac{1}{T}-\frac{1}{Tm})}}$$

where *r_s_* is the average count rate, *T* is the temperature (°C), *p_1_* and *p_2_* correspond to the slopes of the straight lines at the beginning and the end of the plot, and r_s1_ and r_s2_ are the respective count rate intercepting values at the y axis. From the experimental data, it is possible to calculate the cooperativity (B) and the midpoint of the phase transition, which corresponds to *T_m_* [35].

### 4.5. Surface Plasmon Resonance Assays

Peptide-membrane interactions were studied by SPR at 25 °C, using a Biacore sensor chip L1 in a Biacore 3000 instrument (GE Healthcare, Uppsala, Sweden), with an HBS-N buffer (0.01 M HEPES, pH 7.4, 0.15 M NaCl) as a running buffer. POPC and POPG SUV, 50 nm in diameter, were prepared with HBS-N, as previously described, and deposited onto the L1 chip at a flow rate of 2 µL/min for 40 min, reaching a steady-state plateau that confirmed chip surface coverage with deposited lipid bilayers. Several dilutions of TP10 and CQ-TP10 conjugates were prepared in the HBS-N buffer and injected over the lipid bilayer (flow rate of 5 µL/min, 180 s). Peptide-lipid dissociation followed for 600 s [20]. Response units (RU) from sensorgrams were converted to a peptide-to-lipid ratio (P/L, mol/mol), assuming 1 RU = 1 pg.mm^−2^ of lipid or peptide. The maximum P/L (mol/mol) achieved at the end of the association phase (170 s) was calculated to normalize the response to the molecular weight of each peptide and the deposition level achieved for each lipid system to allow for comparison of the binding between peptides and across different lipids. Regeneration of the sensor chip’s surface was achieved upon the injection of an aqueous solution of 3-[(3-cholamidopropyl)dimethylammonio]-1-propanesulfonate hydrate, or CHAPS, (20 mM; flow rate of 5 µL/min, 60 s; two times), followed by the injection of aqueous solutions of HCl 100 mM, NaOH 10 mM with 20% MeOH (*v/v*) and NaOH 10 mM (30 µL; flow rate of 50 µL/min). Finally, CHAPS was once again injected (20 mM; flow rate of 5 µL/min, 60 s) prior to lipid deposition.

## 5. Concluding Remarks

The significant increase in the haemolytic activity of TP10 upon its conjugation with CQ suggests that this type of payload impedes the CPP’s ability to display expected cell penetrating properties that might be useful for the enhancement of antiplasmodial action, eventually also by eluding resistance mechanisms in CQ-resistant parasite strains. Instead, the produced conjugates exert strong membranolytic activity, especially on the zwitterionic membrane models of non-parasitized RBC, as demonstrated herein. This puts a spotlight on the non-universality of CPP as drug carriers, as undesirable cytotoxic effects may rise depending on which drug will be used as payload. Still, according to the disease that is targeted, one may be willing to take the risk: if CPP conjugation is aimed at enhancing the action of, e.g., an antineoplastic agent, increased cytotoxicity may be desirable even if there is some loss in selectivity. In fact, most of literature reports on CPP-drug conjugates concern the targeting of antineoplastic agents and include in vitro activity tests often carried out using cancer cell lines only, which precludes any assessment of selectivity. Hence, a fine scrutiny of CPP-drug conjugates’ action on both normal and altered (diseased) cells, eventually complemented with biophysical assessment of their interactions with their respective lipid membrane models, should be considered as mandatory.

## Data Availability

Please refer to suggested Data Availability Statements in section “MDPI Research Data Policies” at https://www.mdpi.com/ethics.
